# Long‐term overall survival and toxicities of ABVD vs BEACOPP in advanced Hodgkin lymphoma: A pooled analysis of four randomized trials

**DOI:** 10.1002/cam4.3298

**Published:** 2020-07-25

**Authors:** Marc P. E. André, Patrice Carde, Simonetta Viviani, Monica Bellei, Catherine Fortpied, Martin Hutchings, Alessandro M. Gianni, Pauline Brice, Olivier Casasnovas, Paolo G. Gobbi, Pier Luigi Zinzani, Jehan Dupuis, Emilio Iannitto, Alessandro Rambaldi, Josette Brière, Laurianne Clément‐Filliatre, Marian Heczko, Pinuccia Valagussa, Jonathan Douxfils, Julien Depaus, Massimo Federico, Nicolas Mounier

**Affiliations:** ^1^ Hematology Department CHU UCL Namur Yvoir Belgium; ^2^ Institut Gustave Roussy Villejuif France; ^3^ Department of Medical Oncology and Hematology Fondazione IRCCS Istituto Nazionale dei Tumori Milan Italy; ^4^ Division of Hemato‐Oncology IEO European Institute of Oncology Milan Italy; ^5^ Fondazione Italiana Limfomi Dipartimento di Scienze Medische e Chirurgiche Materno‐Infantili e dell'Adulto University of Modena, e Reggio Emilia Modena Italy; ^6^ European Organisation for Research and Treatment of Cancer Brussels Belgium; ^7^ Department of Hematology, Rigshospitalet University of Copenhagen Copenhagen Denmark; ^8^ APHP Hopital Saint‐louis Hemato‐oncologie Université Paris Diderot Sorbonne Paris Cité Paris France; ^9^ Hematology department and INSERM1231 Hôpital F. Mitterand Dijon France; ^10^ Department of Internal Medicine Fondazione IRCCS Policlinico San Matteo and Università degli Studi di Pavia Pavia Italy; ^11^ Institute of Hematology “Seràgnoli” University of Bologna Bologna Italy; ^12^ Unité Hémopathies Lymphoïdes AP‐HP Hôpital Henri Mondor Créteil France; ^13^ Department of Oncology Haematology Unit AOU Policlinico P. Giaccone Palermo Italy; ^14^ Department of Oncology‐Hematology Università degli Studi di Milano e Azienda Socio Sanitaria Territoriale Papa Giovanni XXIII Bergamo Italy; ^15^ Department of Hematology CHU Nancy Nancy France; ^16^ Department of Hematology Besancon Hospital Besancon France; ^17^ Department of Pharmacy Namur Thrombosis and Hemostasis Centre (NTHC) Namur Research Institute for Life Sciences (NARILIS) University of Namur Namur Belgium; ^18^ Department of Surgery, Medicine Dentistry and Morphological Sciences with Transplant Surgery, Oncology and Regenerative Medicine Relevance University of Modena and Reggio Emilia Centro Oncologico Modenese Modena Italy; ^19^ Department of Onco‐Haematology Archet Hospital Nice France

**Keywords:** ABVD, BEACOPP, Hodgkin lymphoma, overall survival, progression‐free survival, secondary cancers

## Abstract

**Purpose:**

We explored the potential overall survival (OS) benefit of bleomycin, etoposide, doxorubicin (Adriamycin), cyclophosphamide, vincristine (Oncovin), procarbazine, and prednisone (BEACOPP) over doxorubicin (Adriamycin), bleomycin, vinblastine, and dacarbazine (ABVD) in a pooled analysis of four randomized trials.

**Patients and methods:**

Primary objective was to evaluate the OS impact of BEACOPP using individual patient data. Secondary objectives were progression‐free survival (PFS), secondary cancers, and use of autologous stem cell transplantation (ASCT).

**Results:**

About 1227 patients were included. The 7‐year OS was 84.3% (95% CI 80.8‐87.2) for ABVD vs 87.7% (95% CI 84.5‐90.2) for BEACOPP. Two follow‐up periods were identified based on survival curves and hazard ratio (HR) over time. For the first 18 months, there was no difference. For the second period of ≥18 months, ABVD patients had a higher death risk (HR_ABVD vs BEACOPP_ = 1.59; 95% CI 1.09‐2.33). A Cox model stratified by trial and evaluating the effect of treatment and International Prognostic Index (IPI) score as fixed effects showed that both were statistically significant (treatment, *P* = .0185; IPI score, *P* = .0107). The 7‐year PFS was 71.1% (95% CI 67.1‐74.6) for ABVD vs 81.1% (95% CI 77.5‐84.2) for BEACOPP (*P* < .001). After ABVD, 25 secondary cancers (4.0%) were reported with no myelodysplasia (MDS)/acute myeloid leukemia (AML) compared to 36 (6.5%) after BEACOPP, which included 13 patients with MDS/AML. Following ABVD, 86 patients (13.8%) received ASCT vs 39 (6.4%) for BEACOPP.

**Conclusions:**

This analysis showed a slight improvement in OS for BEACOPP and confirmed a PFS benefit. Frontline use of BEACOPP instead of ABVD increased secondary leukemia incidence but halved the requirement for ASCT.

## Graphical abstract

Advanced Hodgkin lymphoma (HL) are treated with two different chemotherapy regimens (doxorubicin [Adriamycin], bleomycin, vinblastine, and dacarbazine [ABVD] or bleomycin, etoposide, doxorubicin [Adriamycin], cyclophosphamide, vincristine [Oncovin], procarbazine, and prednisone [BEACOPP]) that have two different toxicity profiles. In this pooled analysis of four randomized trials comparing these two regimens, and with a median follow‐up of 7 years, progression‐free survival is significantly superior with the BEACOPP regimen. The 7 years overall survival was 84.3% for ABVD and 87.7% for BEACOPP. The main cause of death after ABVD is HL, but second malignancy including 10 myeloid malignancies after BEACOPP.

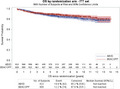

## 1 INTRODUCTION

Hodgkin lymphoma (HL) is one of the most curable adult cancers, with long‐term cure rates of >80% achieved even in patients with advanced disease.[1] The combination of doxorubicin (Adriamycin), bleomycin, vinblastine, and dacarbazine (ABVD) is a current standard of care but is challenged by the German Hodgkin Study Group (GHSG) who developed a combination consisting of bleomycin, etoposide, doxorubicin (Adriamycin), cyclophosphamide, vincristine (Oncovin), procarbazine, and prednisone (BEACOPP). The HD9 trial conducted by the GHSG demonstrated the superiority of BEACOPP compared to cyclophosphamide, vincristine (Oncovin), procarbazine, and prednisone alternating with ABVD (COPP‐ABVD) both in terms of failure‐free survival and overall survival (OS).[2] As COPP/ABVD used in the HD9 trial was not a standard regimen and because of concerns about the toxicity of BEACOPP, several groups have conducted head‐to‐head comparisons of ABVD and BEACOPP.[3, 4, 5, 6, 7] All of these phase III randomized trials showed a benefit for BEACOPP in terms of disease control. However, although the OS estimates were in favor of BEACOPP, they never reached statistical significance in each individual trial. After screening the literature, we confirmed four randomized trials comparing ABVD and BEACOPP. We pooled the individual patient data from these trials to evaluate OS with an increased statistical power and, also, with a longer median follow‐up.

## 2 PATIENTS AND METHODS

### 2.1 Study selection and data extraction

We searched the following databases without any language restrictions: the Cochrane Library including the Cochrane Central Register of Controlled Trials, Medline (Ovid: January 1980‐June 2016), and conference proceedings of annual meetings of the American Society of Hematology, American Society of Clinical Oncology, and European Hematology Association if not included in CENTRAL. Two independent investigators (JDe and JDo) selected studies. Both investigators screened the titles and abstracts of study reports identified by the search strategy for eligibility. Disagreements were resolved by discussion with a third author (MA).

We only included phase III randomized controlled trials in adult patients with newly diagnosed advanced‐stage HL, as defined by the investigators, assessing any of the following prespecified interventions: BEACOPP_baseline_, BEACOPP_escalated_, BEACOPP variants (ie four cycles of BEACOPP_escalated_ [4‐BEACOPP_escalated_] followed by two or four cycles of BEACOPP_baseline_ [2‐BEACOPP_baseline_ or 4‐BEACOPP_baseline_]), and ABVD. Trial reports had to provide data on OS for inclusion irrespective of follow‐up duration.

After this literature review, four individual patient data corresponding to the selection criteria were identified: HD2000,[3, 7] Italian Intergroup on Lymphoma (IIL),[4] H34 low risk,[5] and EORTC20012[6]). In HD2000, patients received six courses of ABVD (6‐ABVD) vs 4‐BEACOPP_escalated_ plus 2‐BEACOPP_baseline_. In IIL, patients received 6‐ABVD vs 4‐BEACOPP_escalated_ plus 4‐BEACOPP_baseline_. In H34 low risk, patients received eight courses of ABVD (8‐ABVD) vs 4‐BEACOPP_escalated_ plus 4‐BEACOPP_baseline_. In EORTC20012, patients received 8‐ABVD vs 4‐BEACOPP_escalated_ plus 4‐BEACOPP_baseline_. Follow‐up was short in the original reports of the studies H34 low risk[5] and EORTC20012,[6] so data were updated for longer follow‐up conducted in March 2017 for these two studies. For the HD2000 study,[3] an update with a longer follow‐up was recently published[7] and was used in our current analysis. The data of the IIL study[4] could not be updated for follow‐up due to regulatory issues.

All patients provided written, informed consent before enrolment in each study. The study was approved the CHU UCL Namur Ethics Committee and was done in accordance with the Declaration of Helsinki and the International Conference on Harmonization Guidelines for Good Clinical Practice.

### 2.2 Study objectives

The primary objective was to evaluate the impact of BEACOPP vs ABVD on OS. Secondary objectives were to compare progression‐free survival (PFS), secondary cancers, causes of death, and use of high‐dose chemotherapy with autologous stem cell transplantation in relapsing or refractory patients. OS and PFS were defined as the time from the date of randomization to the date of death from any cause and to the date of disease progression or death from any cause, respectively.

### 2.3 Statistical analysis

Individual participant data obtained from the authors of the original studies were used for analyses. OS and PFS were analyzed according to randomized treatment arm for the intent‐to‐treat populations. Intent‐to‐treat population was defined as: all randomized patients (even if later found to be ineligible) with informed consent in IIL, H34 low risk, and EORTC200012; and all patients excluding patients for whom an exclusion criterion was discovered after random assignment and patients who had insufficient follow‐up information to determine treatment outcome in HD2000. The safety population was defined as all patients enrolled in the study who started the allocated protocol therapy.

The types of events taken into account for survival analysis were described in numbers and percentages. Survival estimates resulting from Kaplan‐Meier survival analysis and hazard rate over time were presented graphically. Multivariate analysis was performed using a Cox regression model stratified by trial. The median time‐to‐event was estimated with 95% confidence intervals (CIs). In addition, the event rates at specific time points were computed along with 95% CIs. Estimates of the treatment effect were expressed as hazard ratios (HRs) with 95% CIs. All tests were two‐sided and performed at a significance level of 5%.

## 3 RESULTS

The four selected trials recruited 1227 patients (622 ABVD and 605 BEACOPP). Patient characteristics are summarized in Table 1 and are well balanced between the two arms. Median age was 32 years (range 15‐67) with a male predominance (63.9%). A total of 86.9% patients were stage III‐IV; patients with stage II high risk could be included in the IIL and HD2000 studies alone and represented 12.9% of all patients. Overall, 37.8% of the population had bulky disease and 65.9% had International Prognostic index (IPI) score ≥ 3.[8] Median (95% CI) follow‐up for all patients included in this analysis was 7 (6.6‐7.2) years and was similar in both arms. It was 10 (9.3‐10.3) years in the HD2000 study, 5.1 (4.7‐5.2) years in the IIL study, 7.7 (6.5‐8.5) years in the H34 low risk study, and 7.6 (7.1‐8.0) years in the EORTC20012 study.

**TABLE 1:** Patient demographics and baseline disease characteristics (intent‐to‐treat populations)

Characteristic	H34 low risk		EORTC20012		HD2000		IIL		All four studies		
	ABVD (N = 80)	BEACOPP (N = 70)	ABVD (N = 275)	BEACOPP (N = 274)	ABVD (N = 99)	BEACOPP (N = 98)	ABVD (N = 168)	BEACOPP (N = 163)	ABVD (N = 622)	BEACOPP (N = 605)	Total (N = 1227)
Age, y											
Median	28	28	34	35	31	29	32	31	32	32	32
Range	16‐60	16‐58	16‐67	16‐60	17‐64	15‐66	16‐65	16‐60	16‐67	15‐66	15‐67
Gender, n (%)											
Male	42 (52.5)	33 (47.1)	209 (76.0)	204 (70.5)	43 (43.4)	59 (60.2)	100 (59.5)	94 (57.7)	394 (63.3)	390 (64.5)	784 (63.9)
Female	38 (47.5)	37 (52.9)	66 (24.0)	70 (25.5)	5§ (56.6)	39 (39.8)	68 (40.5)	69 (42.3)	228 (36.7)	228 (36.7)	443 (36.1)
Histology, n (%)											
Nodular sclerosis	45(86.5)	49 (90.7)	195 (74.4)	184 (71.0)	80 (80.8)	82 (83.7)	131 (78.0)	132 (81.0)	451 (77.6)	447 (77.9)	898 (77.7)
Mixed cellularity	2 (3.8)	2 (3.7)	37 (14.1)	43 (16.6)	11 (11.1)	7 (7.1)	26 (15.5)	21 (12.9)	76 (13.1)	73 (12.7)	149 (12.9)
Other	5 (9.6)	3 (5.6)	30 (11.4)	32 (12.3)	8 (8.0)	9 (9 0.1)	11 (6.5)	10 (6.1)	54 (9.3)	54 (9.4)	108 (9 0.4)
Missing	28	16	13	15	0	0	0	0	41	31	72
Clinical stage, n (%)											
I	0 (0)	0 (0)	0	0 (0.0)	0 (0.0)	0 (0.0)	1 (0.6)	0 (0.0)	1 (0.2)	0 (0.0)	1 (0.1)
II	0 (0)	1 (1.4)	1 (0.4%)	1 (0.4)	33 (33.3)	30 (30.6%)	46 (27.4)	46 (28.2)	80 (12.9)	78 (12.9)	158 (12.9)
III	48 (60.0)	29 (41.4)	65 (23.6)	76 (27.9)	44 (44.4)	46 (46.9%)	45 (26.8)	43 26.4)	202 (32.5)	194 (32.2)	396 (32.3)
IV	32 (40.0)	40 (57.0)	209 (76.0)	195 (71.7)	22 (22.2)	22 (22.4%)	76 (45.2)	74 (45.4)	339 (54.5)	331 (54.9)	670 (54.6)
Missing	0	0	0	2	0	0	0	0	0	2	2
B symptoms, n (%)											
No	30 (38)	32 (45.7)	55 (20.1)	47 (17.3)	40 (40 0.4)	27 (27.6)	27 (16.1)	25 (15.3)	152 (24.5)	131 (21.7)	283 (23.1)
Yes	49 (62)	38 (54.3)	219 (79.9)	225 (82.7)	59 (59.6)	71 (72.4)	141 (83.9)	138 (84.7)	468 (75.5)	472 (78.3)	940 (76.9)
Missing	1	0	1	2	0	0	0	0	2	2	4
Bulky disease, n (%)											
No	55 (75.3)	53 (81.5)	189 (71.1)	176 (67.7)	68 (68.7)	62 (63.3)	76 (45.2)	62 (38.0)	388 (64.0)	353 (60.2)	741 (62.2)
Yes	18 (24.7)	12 (18.5)	77 (28.9)	84 (32.3)	31 (31.3)	36 (36.7)	92 (54.8)	101 (62.0)	218 (36.0)	233 (39.8)	451 (37.8)
Missing	7	5	9	14	0	0	0	0	16	19	35
Bone marrow involvement, n (%)											
Negative	71 (92.2)	61 (91.0)	198 (76.4)	215 (81.7)	93 (94.9)	81 (85.3)	153 (91.1)	151 (92.6)	515 (85.5)	508 (86.4)	1023 (86.0)
Positive	6 (7.8)	6 (9.0)	61 (23.6)	48 (18.3)	5 (5.1)	14 (14.7)	15 (8.9)	12 (7.4)	87 (14.5)	80 (13.6)	167 (14.0)
Missing	3	3	16	11	1	3	0	0	20	17	37
IPI score, n (%)											
0‐2	69 (97.2)	61 (93.8)	2 (0.7)	2 (0.7)	68 (70.1)	55 (57.3)	79 (47.0)	76 (46.6)	218 (35.7)	194 (32.6)	412 (34.1)
≥3	2 (2.8)	4 (6.2)	273 (99.3)	270 (99.3)	29 (29.9)	41 (42.7)	89 (53.0)	87 (53.4)	393 (64.3)	402 (67.4)	795 (65.9)
Missing	9	5	0	2	2	2	0	0	11	9	20

Note

Because of rounding, percentages may not total 100 exactly.

Abbreviations: ABVD, doxorubicin, bleomycin, vinblastine, and dacarbazine; BEACOPP, bleomycin, etoposide, doxorubicin, cyclophosphamide, vincristine, procarbazine, and prednisone; IPI, International Prognostic Index.

### 3.1 Primary endpoint: OS

**FIGURE 1:**
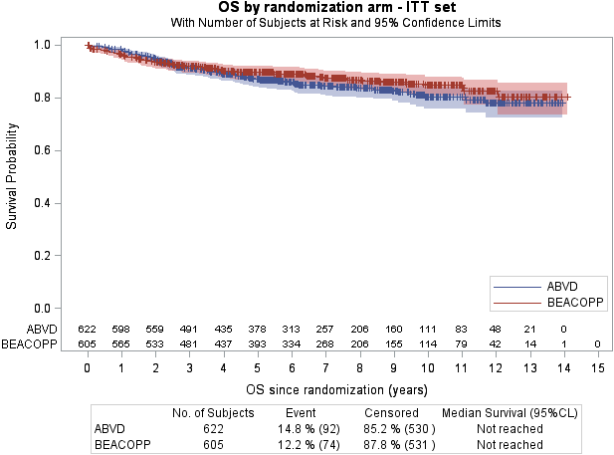
Kaplan‐Meier curve for overall survival of all patients included in the four studies comparing ABVD and BEACOPP. ABVD, doxorubicin, bleomycin, vinblastine, and dacarbazine; BEACOPP, bleomycin, etoposide, doxorubicin, cyclophosphamide, vincristine, procarbazine, and prednisone

OS analysis was performed on the four studies and 166 deaths (13.5%) were reported. The 7‐year OS was 84.3% (95% CI 80.8‐87.2) in the ABVD arm vs 87.7% (95% CI 84.5‐90.2) in the BEACOPP arm; 10‐year OS was 80.2% (95% CI 75.7‐84.0) in the ABVD arm vs 84.7% (95% CI 80.5‐88.0) in the BEACOPP arm (Figure 1). OS by treatment in each study is reported in the appendix (Figures S1‐S4). As HR was not constant over time (Figure 1), two follow‐up periods were identified based on HR over time: ≤18 vs >18 months after randomization. For the first period, patients alive 18 months after randomization were censored at 18 months. For the second period, only patients alive 18 months after randomization were considered.

#### 3.1.1 Less than 18 months follow‐up (N = 1227)

There were 54 deaths (4.4%), with no difference being detected between the treatment arms (HR_ABVD vs BEACOPP_ = 0.715; 95% CI 0.417‐1.226; *P* = .22).

#### 3.1.2 After 18 months follow‐up (N = 1128)

There were 112 deaths (9.9%). Patients in the ABVD arm had a higher risk of death compared to those in BEACOPP arm: HR_ABVD vs BEACOPP_ = 1.592; 95% CI 1.088‐2.33; *P* = .0167). At 5 years, OS was estimated at 88.3% (95% CI 85.0‐90.9) in the ABVD arm and 93.2% (95% CI 90.5‐95.2) in the BEACOPP arm.

Another evaluation performed on two different time period (≤30 vs >30 months) showed similar results (see Appendix S1).

#### 3.1.3 Multivariate analysis

An exploratory analysis of the effect of IPI score was also performed: 412 patients had a score of 0‐2, 797 had a score of ≥3, and 18 had missing data. The 7‐year OS was 92.7% (95% CI 89.5‐95.0) for IPI score 0‐2 and 82.3% (95% CI 79.1‐85.1) for IPI score ≥3, with an HR of 0.415 (95% CI 0.280‐0.613; *P* < .001).

Additionally, a Cox model was constructed stratified by trial to evaluate the effect of treatment and IPI score (0‐2 vs ≥3) as fixed effects. Both treatment and IPI score were statistically significant for the period >18 months after randomization (treatment effect *P* = .0185; IPI score *P* = .0107). Patients treated with ABVD had a higher risk of death than patients treated with BEACOPP (HR_ABVD vs BEACOPP_ = 1.586; 95% CI 1.081‐2.328). A high IPI score (≥3) significantly increased the risk of death (HR_high vs low_ = 2.284; 95% CI 1.212‐4.306).

For the period ≤18 months after randomization, only IPI score was significant (treatment effect *P* = .2485, HR_ABVD vs BEACOPP_ = 0.728, 95% CI = 0.424‐1.249; IPI score *P* = .0192, HR_high vs low_ = 2.811; 95% CI = 1.183‐6.678).

#### 3.1.4 Causes of death

Causes and number of deaths are reported in Table 2. Altogether, 166 (13.3%) patients died, of which 78 (47%) were from progression of HL. In the ABVD arm, 93 patients died: the primary cause of death was HL (n = 57) followed by toxicities (n = 15) and secondary cancer (n = 8). In the BEACOPP arm, 73 patients died: the primary cause of death was secondary cancer (n = 22, including 10 patients with secondary myelodysplasia [MDS]/acute myeloid leukemia [AML]), HL progression (n = 21), and toxicities (n = 16).

**TABLE 2:** Causes of death (intent‐to‐treat populations)

Median follow‐up 95% CI	H34 low risk 7.6 y 6.5‐8.6		EORTC20012 7.7 y 7.1‐8.0		HD2000 10 y 9.3‐10.3		IIL 5.1 y 4.7‐5.2		All four studies 7 y 6.6‐7.2			
	ABVD (N = 77)	BEACOPP (N = 68)	ABVD (N = 272)	BEACOPP (N = 269)	ABVD (N = 107)	BEACOPP (N = 89)	ABVD (N = 168)	BEACOPP (N = 163)	ABVD (N = 624)	BEACOPP (N = 589)	Total (N = 1213)	
Death, n (%)												
No	67 (87.0)	67 (98.5)	225 (82.7)	226 (84.0)	93 (86.9)	75 (84.3)	146 (86.9)	148 (90.8)	531 (85.1)	516 (87.6)	1047 (86.3)	
Yes	10 (13.0)	1 (1.5)	47 (17.3)	43 (16.0)	14 (13.1)	14 (15.7)	22 (13.1)	15 (9.2)	93 (14.9)	73 (12.4)	166 (13.7)	
Cause, n (n of deaths < 18 mo)									n (% of all deaths)		
HL	5 (0)	0 (0)	20 (3)	11 (4)	13 (1)	4 (2)	19 (9)	6 (1)	57 (61.3)	21 (28.8)	78 (47.0)	
Treatment toxicity	1 (0)	0 (0)	11 (6)	7 (6)	0 (0)	1 (0)	2 (1)	8 (6)	14 (15.0)	16 (21.9)	30 (18.1)	
ASCT toxicity	0 (0)	0 (0)	0 (0)	0 (0)	1 (1)	0 (0)	0 (0)	0 (0)	1 (1.1)	0 (0.0)	1 0.6)	
Second cancer	3 (0)	1 (1)	4 (0)	15 (1)	0 (0)	5 (0)	1 (0)	1 (0)	8 (8.6)	22 (30.1)	30 (18.1)	
MDS/AML	0 (0)	0 (0)	0 (0)	8 (0)	0 (0)	1 (0)	0 (0)	1 (0)	0	10	10	
Other cancers	3 (0)	1(1)	4 (0)	7 (1)	0 (0)	4 (0)	1 (0)	0 (0)	8	12	20	
Infection	0 (0)	0 (0)	2 (2)	3 (2)	0 (0)	4 (4)	0 (0)	0 (0)	2 (2.1)	7 (9.6)	9 (5.4)	
Cardiovascular	0 (0)	0 (0)	3 (1)	1 (1)	0 (0)	0 (0)	0 (0)	0 (0)	3 (3.2)	1 (1.4)	4 (2.4)	
Other/unknown	1 (0)	0 (0)	7 (1)	6 (1)	0 (0)	0 (0)	0 (0)	0 (0)	8 (8.6)	6 (8.2)	14 (8.4)	

Abbreviations: ABVD, doxorubicin, bleomycin, vinblastine, and dacarbazine; AML, acute myeloid leukemia; ASCT, autologous stem cell transplantation; BEACOPP, bleomycin, etoposide, doxorubicin, cyclophosphamide, vincristine, procarbazine, and prednisone; HL, Hodgkin lymphoma; MDS, myelodysplastic syndrome.

### 3.2 Secondary endpoints

#### 3.2.1 Progression‐free survival

**FIGURE 2:**
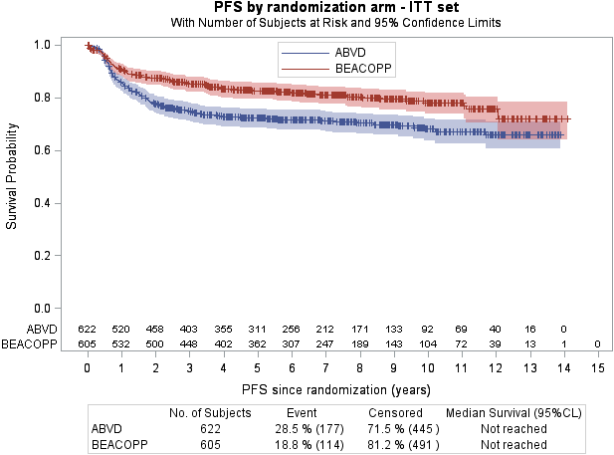
Kaplan‐Meier curve for progression‐free survival of all patients included in the four studies comparing ABVD and BEACOPP. ABVD, doxorubicin, bleomycin, vinblastine, and dacarbazine; BEACOPP, bleomycin, etoposide, doxorubicin, cyclophosphamide, vincristine, procarbazine, and prednisone

A total of 291 patients (23.7%) had an event (progression/relapse [n = 214] and death from any cause [n = 77]). The 7‐year PFS was 71.1% (95% CI 67.1‐74.6) for ABVD vs 81.1% (95% CI 77.5‐84.2) for BEACOPP (*P* < .001) (Figure 2). Patients in the ABVD arm were more likely to relapse, progress, or die than patients in BEACOPP arm (HR_ABVD vs BEACOPP_ = 1.604, 95% CI 1.267‐2.030). PFS by treatment in each study is reported in the appendix (Figures S5‐S8). International Prognostic index score had a significant effect on PFS: the HR of IPI score 0‐2 was 0.706 (95% CI 0.545‐0.913; *P* = .007).

#### 3.2.2 Secondary cancers

Sixty‐three secondary cancers occurred during follow‐up in 61 patients (one patient experienced three secondary cancers). Secondary cancers are reported in Table 3. The incidence of secondary cancers per 1000 person‐years was 6.3% (95% CI 4.29‐9.39) in ABVD arm vs 9.6% (95% CI 4.29‐13.25) in BEACOPP arm. Six cases of solid tumors occurred in patients who also received previous radiotherapy.

**TABLE 3:** Secondary cancers. Sixty‐one patients presented 63 secondary cancers (one patient had three secondary cancers in H34 low‐risk ABVD arm)

	H34 low risk		EORTC20012		HD2000		IIL		All four studies		
	ABVD (N = 77)	BEACOPP (N = 68)	ABVD (N = 272)	BEACOPP (N = 269)	ABVD (N = 107)	BEACOPP (N = 89)	ABVD (N = 168)	BEACOPP (N = 163)	ABVD (N = 624)	BEACOPP (N = 589)	Total (N = 1213)
Secondary cancer (n patients)											
No	70 (90.9)	66 (97.1)	258 (94.9)	244 (90.7)	107 (100.0)	83 (93.3)	164 (97.6)	160 (98.2)	599 (96.0)	553 (93.9)	1152 (95)
Yes	7 (9.1)	2 (2.9)	14 (5.1)	25 (9.3)	0 (0.0)	6 (6.7)	4 (2.4)	3 (1.8)	25(4.0)	36(6.1)	61 (5.0)
Type of cancer (n cancer)											
MDS/AML	0	0	0	10	0	1	0	2	0	13	13
NHL	4	1	4	3	0	1	1	0	9	5	14
Myeloproliferative	0	0	2	0	0	0	0	0	2	0	2
Myeloma	1	0	0	0	0	0	0	0	1	0	1
Lung	1	0	3	4	0	2	0	0	4	6	10
Breast	0	0	0	0	0	0	1	0	1	0	1
Colorectal	0	1	2	1	0	0	0	1	2	3	5
Other/unknown	3^a^	0	3^b^	7^c^	0	2^d^	2^e^	0	8	9	17

Abbreviations: ABVD, doxorubicin, bleomycin, vinblastine, and dacarbazine; AML, acute myeloid leukemia; BEACOPP, bleomycin, etoposide, doxorubicin, cyclophosphamide, vincristine, procarbazine, and prednisone; CNS, central nervous system; ENT: ear, nose, and throat; MDS, myelodysplastic syndrome; NHL, non‐Hodgkin lymphoma.

^a^ Chronic lymphocytic leukemia (n = 1), adenocarcinoma of unknown origin (n = 1), carcinoma of unknown origin (n = 1).

^b^ CNS tumor (n = 1), carcinoma of unknown origin (n = 2).

^c^ CNS tumor (n = 1), ENT cancer (n = 2), non‐melanoma cutaneous cancer (n = 1), esophageal cancer (n = 1), biliary tract cancer (n = 1), carcinoma of unknown origin (n = 1).

^d^ Sarcoma of unknown origin (n = 1), Kaposi's sarcoma (n = 1).

^e^ CNS tumor (n = 1), hepatocarcinoma (n = 1).

Thirteen cases of secondary MDS/AML were reported in the BEACOPP arm compared to none in the ABVD arm. One case of AML occurred in a patient who had also received radiotherapy and one case of MDS occurred in a patient who had undergone ASCT. Twelve non‐HLs occurred: eight in the ABVD arm and four in the BEACOPP arm. Thirty‐three solid tumors were reported: 15 in the ABVD arm and 18 in the BEACOPP arm.

#### 3.2.3 Additional treatments

This analysis could only be performed for 882 patients of the safety set, as data were not available for the IIL study. Additional radiotherapy (planned by protocol in HD2000 or within the 3 months following the end of treatment in H34 studies) after chemotherapy was given to 12.6% and 11.3% of the patients in the ABVD and BEACOPP arms, respectively. High‐dose chemotherapy with ASCT was used as second‐line chemotherapy in 61 of 456 patients (13.4%) treated with ABVD and in 27 of 426 patients (6.3%) treated with BEACOPP.

## 4 DISCUSSION

This analysis compared OS after ABVD vs BEACOPP as initial treatment for advanced HL with a median 7‐year follow‐up for the combined results of four randomized studies. We confirmed better initial disease control with BEACOPP over ABVD (7‐year PFS rate 81.1% vs 71.1%): patients who received ABVD were more at risk of relapse, progression, or death. As a consequence of this, more patients needed salvage treatment including ASCT, considered as a standard of care in this setting,[9] in the ABVD group.

For the analysis of OS, two time periods were defined because HR was not constant over time. During the first 18 months, there was no difference between the regimens. After 18 months, a slight difference in OS emerged and is restricted to patients surviving 18 months. Importantly, the causes of death were different between the treatments. The major cause of death in the ABVD group was HL, representing 61.3% of all deaths. However, the main cause of death in the BEACOPP arm was secondary cancer. All 13 cases of secondary MDS/AML reported in this study occurred in the BEACOPP arm and 10 patients died from this complication.

Eichenauer et al[10] have reviewed MDS/AML as a therapy‐related complication, showing patients who received four courses of BEACOPP_escalated_ had an increased risk of developing AML/MDS compared to those treated with less than four cycles of BEACOPP_escalated_ or no BEACOPP chemotherapy (1.7% vs 0.7% vs 0.3%, *P* < .0001). The 2.2% incidence of MDS/AML in our study, which is slightly higher than that reported by the GHSG, emphasizes the need to reduce the toxicity of this regimen. The eight cycles of BEACOPP used in the present analysis is no longer used following the report by Engert et al[11] that six cycles resulted in lower immediate toxicity and improved OS compared to eight cycles when used in combination with positron emission tomography (PET)‐guided radiotherapy. A PET‐guided approach has been evaluated by Borchmann et al,[12] who randomized patients who were PET negative after two cycles of BEACOPP_escalated_ to receive either four or two additional BEACOPP_escalated_ cycles (a total of four BEACOPP_escalated_ cycles in the experimental arm). The study showed that, in PET2‐negative patients, treatment can be reduced to four cycles of BEACOPP_escalated_ without loss of tumor control and 3‐year OS was 98.8% (95% CI 97.2‐100.0) in the 4‐BEACOPP_escalated_ arm. Five patients died from secondary MDS/AML in the standard arm compared to one patient in the experimental 4‐BEACOPP_escalated_ arm. More recently, Casasnovas et al[13] also randomized PET2‐negative patients between 6‐BEACOPP_escalated_ v 2‐BEACOPP_escalated_ plus 4‐ABVD. The primary objective of the study was to show the noninferiority of the 2‐BEACOPP_escalated_ plus 4‐ABVD arm was met. The 5‐year PFS was 86.2% (95% CI 81.6‐89.8) in the standard treatment group vs 85.7% (95% CI 81.4‐89.1) in the PET‐guided treatment group (HR, 1.084; 95% CI 0.737‐1.596; *P* = .65). With a median follow‐up of 50 months, one secondary AML/MDS was reported in each group.

Moving away from BEACOPP, disease control by ABVD could be improved by the addition of new drugs such as brentuximab vedotin or checkpoint inhibitors (eg nivolumab, pembrolizumab). Combination of doxorubicin (Adriamycin), vinblastine, and dacarbazine (AVD) plus brentuximab vedotin has been evaluated by Connors et al[14] in a phase III randomized study for stage III‐IV HL. The 2‐year modified PFS rate for ABVD with and without brentuximab vedotin was 82.1% (95% CI 78.8‐85.0) and 77.2% (95% CI 73.7‐80.4), respectively, a difference of 4.9% points. There was no significant difference in OS at 2 years. Neutropenia, febrile neutropenia, and peripheral neuropathy were more frequent in the AVD plus brentuximab arm. The combination of AVD plus nivolumab was recently evaluated in the Checkmate 205 study and showed an objective response rate of 84% and a 9‐month PFS of 92%.[15] A randomized study comparing AVD plus nivolumab and AVD plus brentuximab vedotin is expected to start recruitment by the end of 2019 (NCT03907488). Further improvement in disease control was more recently evaluated by PET‐guided therapy after ABVD with the aim to reduce the burden of treatment, and therefore, toxicities related to bleomycine. Indeed, the response to the first two cycles of ABVD was reported as a major prognostic factor in advanced HL.[16] Johnson et al[17] showed that PET negative patients after two ABVD had a similar outcome when bleomycin was omitted in the next four cycles. In the same study, PET‐positive patients after two cycles of ABVD had improved PFS (67.5%) and OS (87.8%) when intensified with BEACOPP, which compares favorably with historical controls.

Using these PET‐adapted approaches have reduced both ABVD and BEACOPP toxicities as given in the four randomized studies used for the current analysis.

In conclusion, the current pooled analysis of four randomized studies comparing ABVD vs BEACOPP did not show a clinically meaningful difference in OS in favor of the BEACOPP regimen. Death from HL remains the main concern with ABVD. The major cause of death following BEACOPP was secondary cancer, with secondary MDS/AML being responsible of one‐third of the deaths from secondary cancer. Admittedly, the 7 years of follow‐up achieved in the current study remain still short when late toxicities are concerned and an even more prolonged follow‐up of these studies will be needed to explore the final impact of BEACOPP and ABVD on fertility, secondary cancers, and OS.

## CONFLICT OF INTEREST

The authors do not have any relevant conflict of interest to disclose.

## AUTHOR CONTRIBUTION

Conceptualization: MA, PC, SV, MF, NM; data curation: all; final analysis: MA, PC, CF, SV, MF, NM; writing‐original draft: MA, NM; writing‐review: MA, PC, CF, SV, MF, NM.

## TWEET

ABVD vs BEACOPP in advanced HL. BEACOPP improved PFS, reduced use of ASCT but increased incidence of secondary MDS/AML. Seven years overall survival: 84.3% for ABVD and 87.7% for BEACOPP.

## Supporting information

Supplementary Material
