# Supplementary material for: Long‐term overall survival and toxicities of ABVD vs BEACOPP in advanced Hodgkin lymphoma: A pooled analysis of four randomized trials
Source: Cancer Med. 2020 Jul 25;9(18):6565–75. doi: 10.1002/cam4.3298 (PMC7520354; doi:10.1002/cam4.3298)

**Appendix**

**Long-term overall survival and toxicities of ABVD versus BEACOPP in advanced Hodgkin lymphoma: a pooled analysis of four randomized trials**

**Authors:** Marc André, Patrice Carde, Simonetta Viviani, Monica Bellei, Catherine Fortpied, Martin Hutchings, Alessandro M. Gianni, Pauline Brice, Olivier Casasnovas, Paolo G. Gobbi, Pier Luigi Zinzani, Jehan Dupuis, Emilio Iannitto, Alessandro Rambaldi, Josette Brière, Laurianne Clément-Filliatre, Marian Heczko, Pinuccia Valagussa, Jonathan Douxfils, Julien Depaus, Massimo Federico, Nicolas Mounier

**Supplementary text**

*Less than 30 months follow-up (N = 1227)*

There were 87 deaths (7.1%), with no difference being detected between the treatment arms (HR_ABVD vs BEACOPP_ = 0.957; 95% CI, 0.649–1.505, *P* = 0.97).

*After 30 months follow-up (N = 1026)*

There were 79 deaths (7.7%). Patients in the ABVD arm had a higher risk of death compared to those in BEACOPP arm: HR_ABVD vs BEACOPP_ = 1.551; 95% CI, 0.987–2.437, *P* = 0.057). At 5 years, OS was estimated at 90.6% (95% CI, 87.3–93.1) in the ABVD arm and 94.6% (95% CI, 91.9–96.5) in the BEACOPP arm.

Fig. S1. OS by treatment in the H34 low risk study.


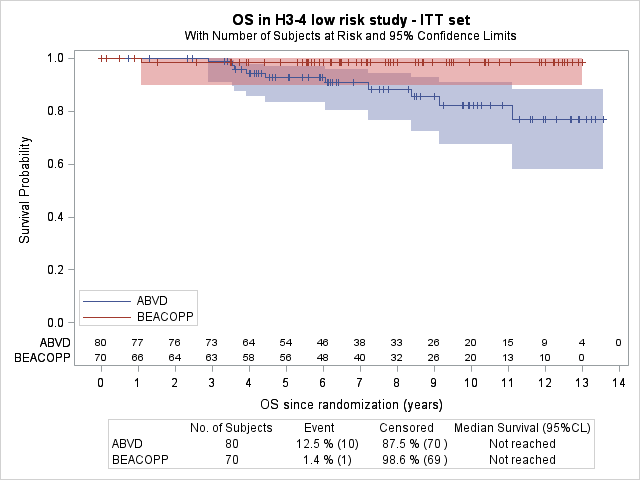


Fig. S2. OS by treatment in the EORTC20012 study.


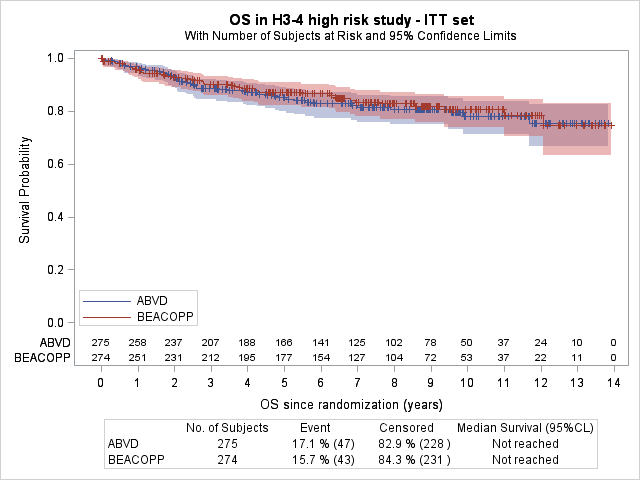


Fig. S3. OS by treatment in the HD2000 study.


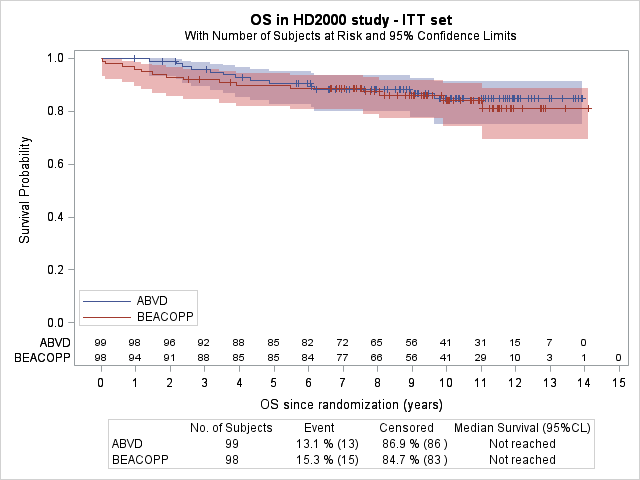


Fig. S4. OS by treatment in the IIL study.


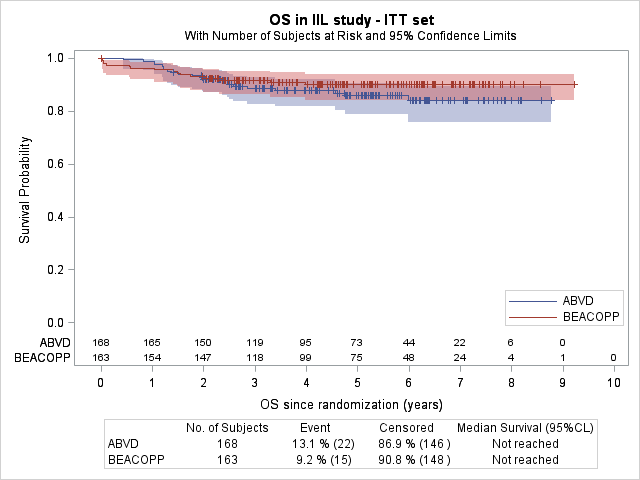


Fig. S5. PFS by treatment in the H34 low risk study.


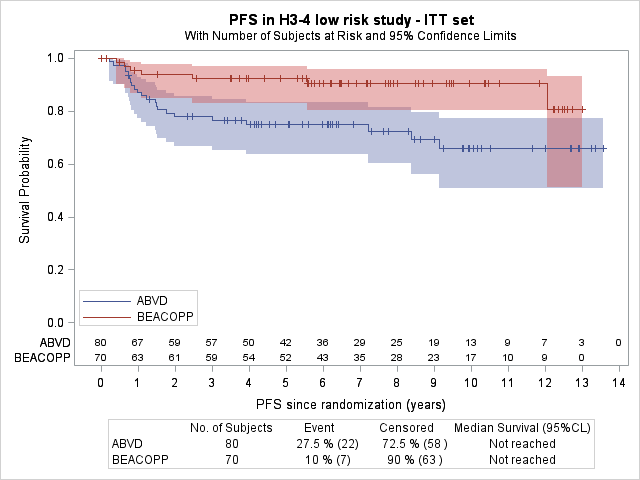


Fig. S6. PFS by treatment in the EORTC20012 study.


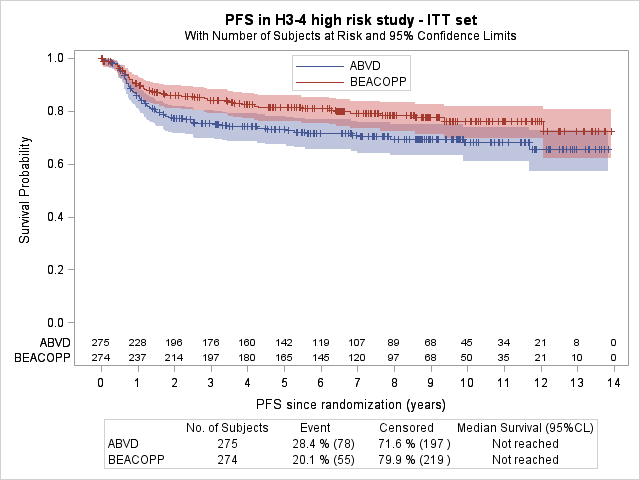


Fig. S7. PFS by treatment in the HD2000 study.


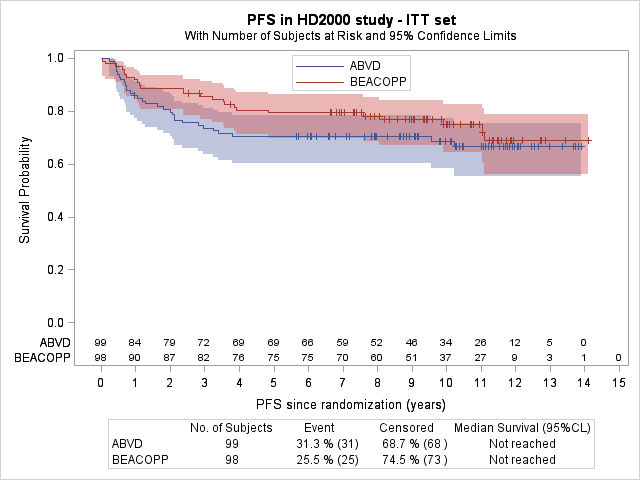


Fig. S8. PFS by treatment in the IIL study.


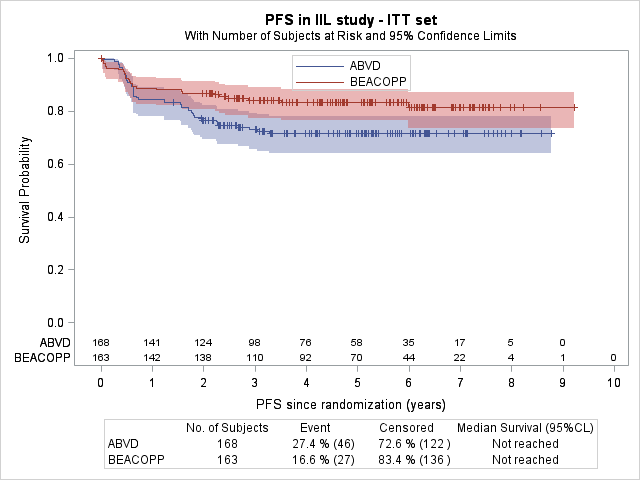

Supplement: Supplementary file 1 — Supplementary Material [file CAM4-9-6565-s001.docx]
